# Retracted: Nontraumatic Lesions of the Clavicle in a Paediatric Population: Incidence and Management

**DOI:** 10.1155/2015/280316

**Published:** 2015-03-25

**Authors:** International Scholarly Research Notices

The paper titled “Nontraumatic Lesions of the Clavicle in a Paediatric Population: Incidence and Management” [1], has been retracted upon receiving the submitting author's confirmation that the paper was submitted without including Dr. Iain Campbell's name among the paper's authors, despite his contribution to the scientific content of the paper.
